# CRISPR based therapeutics: a new paradigm in cancer precision medicine

**DOI:** 10.1186/s12943-022-01552-6

**Published:** 2022-03-25

**Authors:** Sumit Das, Shehnaz Bano, Prachi Kapse, Gopal C. Kundu

**Affiliations:** 1 National Centre for Cell Science, S P Pune University Campus, Pune, 411007 India; 2 School of Basic Medical Sciences, S P Pune University, Pune, 411007 India; 3 Kalinga Institute of Medical Sciences (KIMS), KIIT Deemed To Be University, Bhubaneswar, 751024 India; 4 School of Biotechnology, KIIT Deemed To Be University, Bhubaneswar, 751024 India

**Keywords:** Clustered regularly interspaced short pallindromic repeat (CRISPR), CRISPR-associated protein (Cas), Cas9, Cas12a, CRISPRa, CRISPRi, Precision cancer medicine, CRISPR screen, Nanoparticles, Recombinant viral vectors

## Abstract

**Background:**

Clustered regularly interspaced short palindromic repeat (CRISPR)-CRISPR-associated protein (Cas) systems are the latest addition to the plethora of gene-editing tools. These systems have been repurposed from their natural counterparts by means of both guide RNA and Cas nuclease engineering. These RNA-guided systems offer greater programmability and multiplexing capacity than previous generation gene editing tools based on zinc finger nucleases and transcription activator like effector nucleases. CRISPR-Cas systems show great promise for individualization of cancer precision medicine.

**Main body:**

The biology of Cas nucleases and dead Cas based systems relevant for in vivo gene therapy applications has been discussed. The CRISPR knockout, CRISPR activation and CRISPR interference based genetic screens which offer opportunity to assess functions of thousands of genes in massively parallel assays have been also highlighted. Single and combinatorial gene knockout screens lead to identification of drug targets and synthetic lethal genetic interactions across different cancer phenotypes. There are different viral and non-viral (nanoformulation based) modalities that can carry CRISPR-Cas components to different target organs *in vivo*.

**Conclusion:**

The latest developments in the field in terms of optimization of performance of the CRISPR-Cas elements should fuel greater application of the latter in the realm of precision medicine. Lastly, how the already available knowledge can help in furtherance of use of CRISPR based tools in personalized medicine has been discussed.

## Introduction

**C**RISPR-Cas systems constitute a versatile collection of gene editing tools [1]. Cas effector proteins are RNA-guided endonucleases which can cleave target DNA in controlled fashion. DNA double strand breaks (DSBs) inflicted by Cas nucleases can result in insertion-deletion (indel) mutation or replacement of genomic element depending on whether non-homologous end joining (NHEJ) or homology directed repair pathway (HDR) is chosen for DNA break repair [2, 3]. Moreover, function of Cas nuclease can be multiplexed by simultaneous expression of different RNA species. Nuclease-defective or dead Cas (dCas) nucleases retain capacity to bind DNA and thus are purposed for recruitment of transcription regulators and epigenetic modifiers. CRISPR systems also allow for high throughput screening of phenotypes by parallel perturbation of genetic elements in a pool.

The idea of precision cancer medicine is individualized clinical management of the disease. This requires elucidation of the individual specific genotypes, mapping of synthetic lethal gene interactions and identification of drug targets [4, 5].

In this review, the biology of CRISPR-Cas systems and the diversity of the CRISPR toolbox have been discussed. The focus would be on the CRISPR based genetic screening platforms and the delivery modalities which are the themes of precision medicine applications of CRISPRs [6]. Finally, our perspectives on addressing the current limitations of the CRISPR systems and therapeutic vectors have been highlighted.

### The biology of CRISPR-Cas systems

**Fig. 1:**
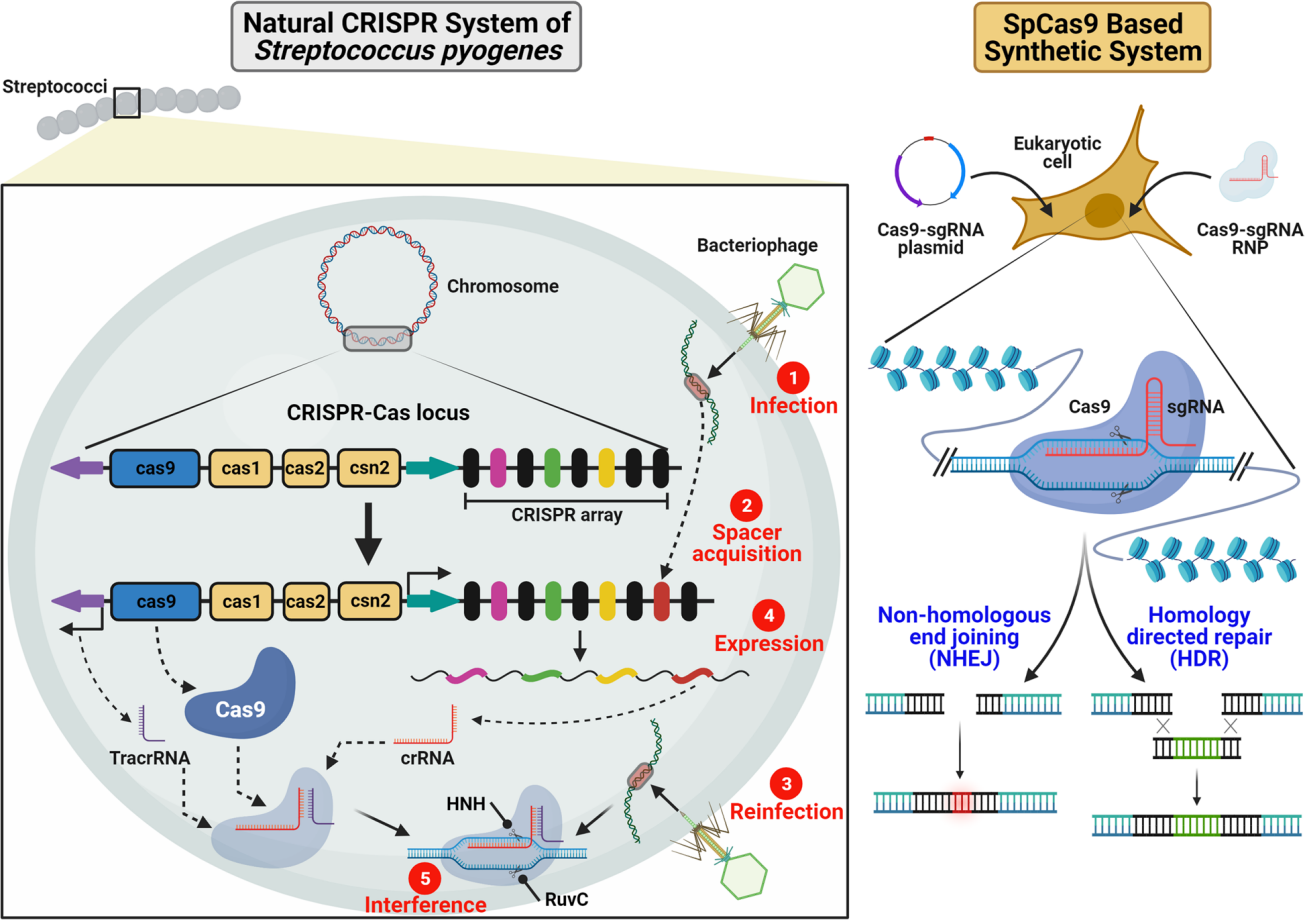
Biology of natural CRISPR-Cas9 system of *Streptococcus pyogenes* and its synthetic counterpart. As the bacteriophage infects a bacterial cell, pieces of phage derived DNA (known as spacers) are inserted in the CRISPR array within CRISPR Cas locus. Following the event of reinfection, the CRISPR array is transcribed. A trans-activating CRISPR RNA (tracrRNA) is also transcribed. CRISPR RNA (crRNA):tracrRNA complexes are derived by the activity of RNase III. The spacer sequence is targeted by the crRNA:tracrRNA:Cas9 complex and cleaved. In case of synthetic systems, cleavage of target sequence is achieved by the activity of single guide RNA (sgRNA)-Cas9 complex. Double strand break is repaired either by the non-homologous end joining (NHEJ) or homology directed repair (HDR) mechanism. Created with BioRender.com with granted permission and license

The CRISPR-Cas9 technology is an excellent example of repurposing of a naturally occurring system for development of a synthetic tool kit for scientific investigations. The CRISPR systems are adaptive immune systems found in bacteria and archaea [7]. They provide immunity to recurrent attacks by bacteriophages, the viruses that attack bacteria. Although recently the CRISPR systems have been characterized from bacteriophages as well [8]. These systems provide the bacteriophages with selective advantage in the process of establishing super infection. Generally, three stages are involved in the CRISPR based immunity: adaptation, expression, and interference [9, 10] (Fig. 1). Since CRISPR systems provide sequence specific immunity, pieces of DNA of the invading bacteriophage known as protospacers are incorporated between the direct repeats of the CRISPR locus. When the bacteriophage attempts at establishing a new infection, the protospacers are transcribed as parts of a larger transcript and processed to produce CRISPR RNAs (crRNAs). The crRNA forms a complex with a trans-activating CRISPR RNA (tracrRNA) and either a multi-component or a single protein effector complex. The crRNA can base-pair with both the target DNA and the tracrRNA. TracrRNA interacts with both the crRNA and the effector protein. Some CRISPR systems lack the participation of a tracrRNA in the final effector complex. In the interference stage, the complex loads on the foreign DNA as the crRNA hybridizes with its complementary sequence. The effector protein subunits are all encoded by the CRISPR locus.

Since it is difficult to categorize the CRISPR-Cas systems based on a single criterion, a “polythetic” approach has been adopted for the same. In this approach, evidence from structural, genomic, and phylogenetic studies have been incorporated. Naturally occurring CRISPR systems are categorized in two broad classes, class 1, and class 2 [9–11]. Each class is composed of several types and subtypes. The effector function is performed by either multiple proteins or a single protein for class 1 and class 2 systems respectively. Mostly the class 2 systems (type II Cas9 and type V Cpf1/Cas12a) have been adopted for gene editing applications. The type II Cas9 system is regarded as the prototype of CRISPR-Cas systems. The biology of *Streptococcus pyogenes* CRISPR-Cas9 (SpCas9) system is well explained [12–14] (Fig. 1). The mature crRNA:tracrRNA complex of this system is formed by the activity of RNase III (which is not encoded by the CRISPR locus). The crRNA guided binding of Cas9 over the target DNA is followed by R loop formation and endonuclease activity. The SpCas9 protein (1368 amino acids) has two nuclease domains, HNH and RuvC domains. The target DNA strand complementary to the CRISPR RNA is cleaved by the HNH endonuclease activity, whereas, the non-complementary strand is cut by the RuvC domain (Fig. 1). A 3-nt sequence (5’-NGG-3’) immediately adjacent (3’ side) to the target sequence is necessary for stable binding and activity of SpCas9. The former is known as protospacer adjacent motif (PAM). Although in the synthetic systems, the tracrRNA is expressed in continuation with the crRNA. Thus, the function of crRNA:tracrRNA complex is performed by a *s*ingle *g*uide *RNA* (sgRNA). The other representative of this type, Cas9 from *Staphylococcus aureus* (SaCas9) (1053 amino acids) has a similar domain architecture although the PAM (5’-NNGRRT-3’) requirement is different [15]. The type V CRISPR-Cas12a system of *Francisella novicida* (FnCas12a) is also well characterized [16–18]. Other Cas12a enzymes have been described from *Acidaminococcus sp*. and *Lachnospiraceae bacterium* and they are known as AsCas12a and LbCas12a [16, 19]. The key difference between Cas9 and Cas12a is that Cas12a can itself process the pre-crRNA to produce mature individual crRNAs [20]. Moreover, CRISPR-Cas12a systems do not encode the tracrRNA. The Cas12a has only one nuclease domain, the RuvC which is composed of three discontinuous stretches of the same polypeptide. The Cas12a systems have a different PAM requirement as compared to Cas9 systems. The Cas12a enzyme recognizes AT-rich PAMs, and these PAMs are situated on the 5’ side of the target sequence. The PAM sequence recognized by FnCas12a is 5’-TTN3’ whereas both AsCas12a and LbCas12a recognize the PAM 5’-TTTN-3’. All these Cas12a orthologs have been demonstrated to have robust activity in human cells.

Synthetic Cas9 and Cas12a based systems can be used to target genes, and cis-regulatory elements (CREs) like enhancers and promoters. The Cas9 systems were described earlier than the Cas12a systems and used widely for the purpose of genome editing. Although in recent years, the Cas12a systems have become more popular for multiplexed genome editing applications. For synthetic Cas9 systems, the expression of multiple sgRNAs are not driven by the same promoter. Since the Cas12a enzymes possess intrinsic RNase activity for processing of the poly-crRNA transcript, these systems are better suited for multiplexed genome editing.

### The CRISPR toolbox

**Fig. 2:**
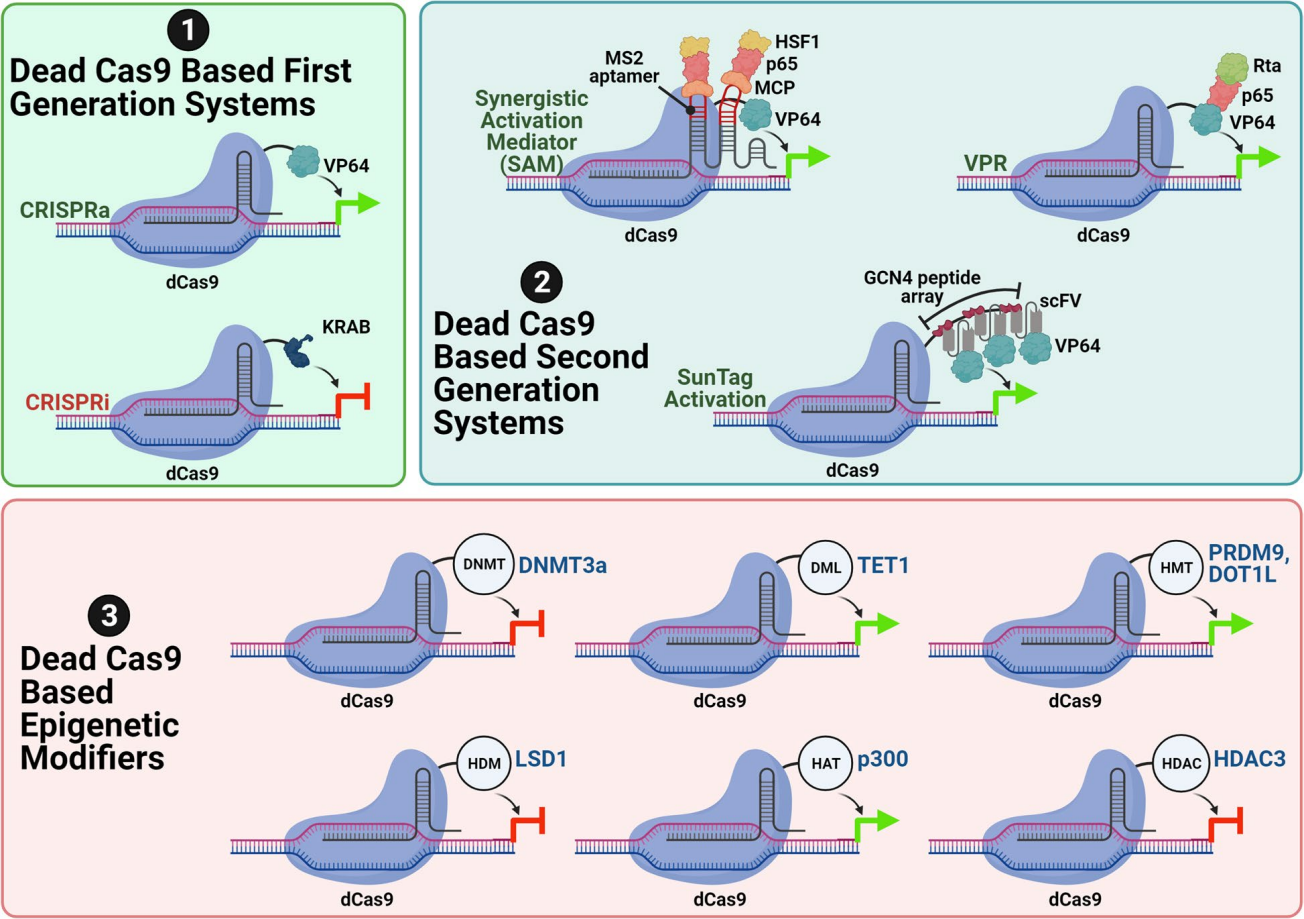
CRISPR toolbox. First and second generation CRISPRa and CRISPRi systems have been illustrated. The second generation CRISPRa systems include the synergistic activation mediator (SAM), the VPR and the SunTag systems. The CRISPR epigenetic modifiers are dead Cas9 based tools that catalyze methylation/demethylation and acetylation/deacetylation of DNA and histones. Created with BioRender.com with granted permission and license

Although the CRISPR gene editing era started off with making wild-type Cas9-mediated gene knockouts (KOs) in different cell biology systems, its versatility was experienced with the derivation of nickase and dCas9. The nickase version of SpCas9 can be obtained by omitting the endonuclease activity of either the HNH (H840A mutation) or the RuvC (D10A mutation) domain [14, 21]. In that case, the nickase can cleave either the target DNA strand complementary to the guide RNA or the opposite strand. A pair of nickases can be used to create a controlled deletion in the target DNA. Nuclease-null or dCas9 has been generated by abolishing the endonuclease activity of both its domains [21]. The dCas9 still acts as a DNA binding protein. Wild-type Cas9 and dCas9-mediated tools have proven to be indispensable for interrogation of complex biological processes in recent years. Programmability of dCas9 by appending different effector molecules rendered the same suitable for several cell biology applications. A major use of dCas9 has been in transcription regulation of endogenous loci in different eukaryotic model systems. Dead Cas9 when appended with either a transcriptional activator or a repressor domain acts to stimulate or inhibit the process of transcription of genetic elements (CRISPR activation/CRISPRa and CRISPR interference/CRISPRi) respectively. The dCas9-based toolbox for transcription regulation has evolved through time with the systems loosely classified as first-generation, second-generation, and third-generation systems [22] (Fig. 2). In the first-generation systems, single transcription activator (like single or multiple copies of viral protein 16 of Herpes Simplex virus; VP16 or VP64) or repressor (like Kruppel associated box/KRAB of Kox1, chromo shadow/CS domain of HP1α) module has been fused with dCas9 [23, 24]. Whilst in the second-generation systems, recruitment of activator or repressor has been augmented by different protein (MS2 coat protein, peptide arrays) or RNA (MS2 stem-loop structure) scaffolds. Different second-generation systems for transcription activation have been devised, namely dCas9-synergystic activation mediator (SAM), dCas9-VP64-p65-Rta (VPR) and dCas9-SunTag-scFv-VP64 [25–27]. In the SAM system, the dCas9 is expressed as a fusion with VP64 transcription activation domain. The guide RNA scaffold is extended to include MS2 stem-loop. Another fusion polypeptide of MS2 coat protein (MCP), p65 minimal activation domain and heat shock factor 1 (HSF1). In the dCas9-VPR and dCas9-SunTag-scFv-VP64 systems, the dCas9 is expressed as fusion with the VP64-P65-Rta and a general control non-derepressible 4 (GCN4) peptide repeat array respectively. In the SunTag system, the activator module is assembled as the GCN4 repeats that are bound by the scFV-VP64 molecules. The third-generation systems have been devised with the idea of temporal and spatial control of transcription regulation. For CRISPRa or CRISPRi, the guide RNAs are designed in such a way that they target a sequence close to the transcription start site (TSS) of a gene. CRISPR-based epigenetic modifiers have been developed by fusion of different histone/DNA methyltransferases, histone/DNA demethylases, histone acetyltransferases and histone deacetylases [28–33]. These epigenetic modifiers bring about histone/DNA post-translational modification, thus affecting contexts for gene regulation (Fig. 2).

Like dead Cas9, a DNase-dead version of Cas12a (ddCas12a) has also been created by mutating critical catalytic site residue of *Acidaminococcus* sp. Cas12a (AsCas12a) [16, 34]. Dead Cas12a mediated CRISPRi machinery has been found to perform better than SpCas9 based CRISPRi system at the same target site [35].

### Precision cancer medicine through the lens of CRISPR

The objective of personalized cancer medicine is individualization of: (i) screening of drug targets and synthetic lethal genetic interactions and (ii) design of therapeutic regimens [4, 5]. Since the genetic and epigenetic landscapes of cancer patients differ, they exhibit different transcriptome, proteome, and metabolome even for the same cancer type. Hence, cancer patients respond differently to the same therapeutic regimen. Over time, resistance to therapy ensues. The difference in mutation landscape and copy number variation (CNV) of driver oncogenes between individuals affects the evolution of resistant cell clones. The latter dictates the duration of response to targeted therapies. Even spatial and temporal intra-tumoral heterogeneity necessitates the primary and metastatic tumors to be treated with different therapeutic regimens [36]. The advent of CRISPR-Cas based techniques provides niche for personalized screening of genetic elements i.e., genes and enhancers those contribute to different facets of cancer progression and subsequent formulation of therapeutics. Such a scope is unprecedented as no other bioengineering tool can aid in massively parallel screening of genotypes and resultant phenotypes. Thus, evolution of the CRISPR toolbox would only broaden the scope of precision cancer medicine. Although zinc finger nuclease (ZFN) and transcription activator-like effector nuclease (TALEN)-based gene editing tools preceded the CRISPRs in development, their biology does not permit use of these tools in high throughput screening applications [37, 38].

### Genome or subgenome wide CRISPR screens in identification of personalized drug targets

Genome or subgenome-wide (subpool) CRISPR screening is one of the few high throughput means of assaying contribution of hundreds or thousands of genetic elements (protein-coding gene, miRNA or long noncoding RNA gene or enhancer) in parallel [39–45]. Genome-wide or sub-pool CRISPR screens are implemented using guide RNA libraries. A guide RNA library is a pool of multiple sets of guide RNAs. Each guide RNA set targets an annotated genetic element in the genome. Cas9 expression is achieved either by incorporation of its expression cassette in the sgRNA backbone or a separate plasmid is used. If all the genetic elements (of a category) in a genome is targeted, the screen is called as genome-wide screen. Whilst screens targeting only a subset of genetic elements (of a category) like oncogenes, tumor suppressor genes, angiogenic genes, metastatic genes or stemness genes are known as subpool screens. The guide RNAs are as members of an oligo pool and the latter is cloned in a suitable vector backbone. The resultant plasmid pool is amplified in a bacterial system. Mostly lentiviral backbones are used for cloning of guide RNA pools to be used in genome-wide or sub-pool screening. Guide RNA libraries are available for genome-wide screening using Cas9 and Cas12 systems [46]. The lentiviruses (LVs) are used to infect the pool of cells to be assayed. The representation of guide RNAs in the plasmid pool or the pool of cells initially infected with LVs is determined by next generation sequencing (NGS). The cells are subjected to the selection pressure relevant to the screen. The guide RNA representation in the final pool is determined using NGS. The relative guide RNA abundance is analyzed to draw logical inferences. Single cell CRISPR screening can also be combined with transcriptome (RNA-seq) or open chromatin sequencing (ATAC-seq) to decipher perturbation associated changes in the RNA pool and chromatin landscape respectively [47–50]. CRISPR screens have been utilized by many in the recent years for identification of genes that contribute to several hallmarks of cancer progression including primary tumor growth, drug resistance, epithelial-to-mesenchymal transition, cancer stemness, metabolic adaptations and metastasis [51–60]. Genome-wide CRISPR screens have also been employed to screen for regulators of genetic dependencies or synthetic lethal gene interactions/drug targets in different cancers [61–66]. Synthetic lethal gene pairs are identified employing double or multiple knockout gRNA libraries. These screens can be performed with either Cas9 or Cas12a. Several Cas12a mutants have been engineered and expression cassette for Cas12a and crRNA have been optimized for maximal performance in combinatorial genetic screens [67, 68]. CRISPRa and CRISPRi based genome-wide or subpool screens can also be performed. Most of these screens till date have been performed either in cell lines using in vitro or transplantation based in vivo models. These bulk and single cell screens can be exercised with patient derived organoids (PDOs) and patient derived xenografts (PDXs) to model patient specific response to therapeutic regimens, metastatic heterogeneity and moreover identify individualized drug targets.

### Delivery of CRISPR Therapeutics

**Fig. 3:**
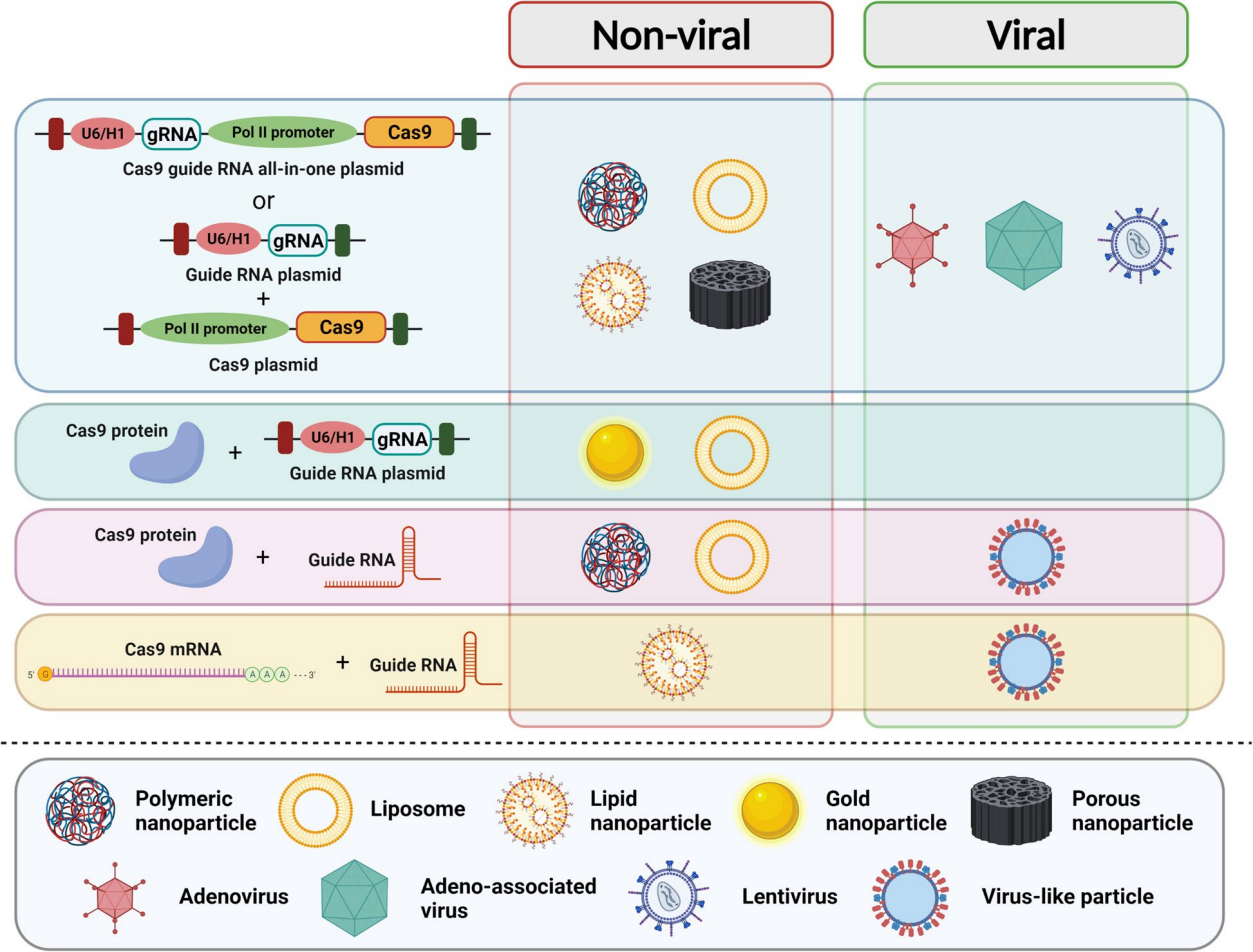
Non-viral and viral vectors for delivery for CRISPR therapeutics. Different non-viral and viral vector systems characterized till date for in vivo delivery of CRISPR based therapeutic agents have been depicted. The non-viral vectors are different nanoformulations like polymeric nanoparticles, lipid nanoparticles, gold nanoparticles and porous nanoparticles. The collection of viral vectors includes adenoviruses, adeno-associated viruses, lentiviruses and virus-like particles. The icons are representative only. Created with BioRender.com with granted permission and license

For therapeutic use of CRISPR based tools, one should ensure that the components are delivered to the target tissue/organ efficiently. Both efficacy and safety of CRISPR therapeutics depend on successful delivery in vivo. Cas nucleases, CRISPRa or CRISPRi systems have been used to target genes or enhancers in preclinical cancer models. The CRISPR-Cas components can be delivered in different physical forms in vivo, as plasmids or recombinant viral genomic DNA or ribonucleoproteins (RNPs). Different non-viral and viral vectors can be utilized for delivery of therapeutic CRISPR Cas systems [69] (Fig. 3). Plasmids encoding CRISPR Cas components are delivered by both the viral systems as well as different nanoparticle (NP) formulations. Cas RNPs are complexes of the Cas enzyme and the guide RNA. These RNPs can also be delivered using NPs. In some cases, the Cas9 mRNA (along with sgRNA) has also been delivered.

Different nanoparticle formulations can be used to shuttle CRISPR components *in vivo* [70]. NP mediated form greatly reduces safety concerns and more precise control can be exerted. Different nanoformulations used for gene and protein delivery include polymeric NPs, liposomes, lipid NPs, gold NPs, and porous NPs. Polymeric and lipid NPs are mostly used for CRISPR gene therapy because of multiple advantages like low immunogenicity, payload protection, good bioavailability. Since the surface of gold nanoparticles (AuNPs) is highly functionalizable, CRISPR nanoformulations can be synthesized by layer-by-layer conjugation of components [71]. Moreover, AuNPs are less toxic than polymeric and lipid nano-carriers.

The viral vectors are good propositions for in vivo delivery as they offer higher efficacy and long-term transgene expression. Mostly, LVs and adeno-associated viruses (AAVs) have been widely investigated for gene therapy applications. Although the approach of virus mediated delivery of transgenes suffers from few limitations. Genome integrating nature of LV vectors poses threat to the safety of CRISPR based therapies as long residence time may lead to greater off target editing. Thus, integrase deficient lentiviral vectors (IDLVs) have been engineered by abolishing the catalytic activity of the integrase enzyme and these are safer options than integrase competent lentiviral vectors (ICLVs) for gene therapy as they ideally have no genomic footprint [72, 73].

Recombinant AAVs have been used as delivery platforms in many instances of in vivo gene targeting. These viruses are capable of packaging of about 5 kb single stranded DNA. Apart from the inverted terminal repeats (ITRs) which are 148 bp in length, rest of the elements can be disrobed. Thus, a total of 4.7 kb long DNA can be packaged between the two ITRs of recombinant AAVs (rAAVs). Assembly of CRISPR components has undergone evolution to make the rAAVs safer and more efficacious for in vivo applications. Although initially a helper vector based self-inactivating circuit was designed, a compact single vector self-limiting rAAV system was developed later although with a PAM-restricted ortholog of Cas9, Nme2Cas9 [74, 75]. Recombinant AAVs are very useful for gene therapy applications as the serotypes preferentially transduce different cell types in the human body i.e., AAV serotypes exhibit differential tropism.

The latest addition to the list of CRISPR delivery platforms are virus-like particles (VLPs) [76]. These resemble the natural viruses but are devoid of the viral genome. Assembly and release of these particles require the activity of only the Gag polypeptide. One advantage offered by VLPs regarding delivery of Cas9 is the transient expression of the latter in the target cells. It minimizes the risk of untoward off target effects. Since the Cas9 coding sequence is not shuttled as part of viral genome, the possibility of insertional mutagenesis is also nullified. These qualities make the VLPs attractive vectors for CRISPR based gene therapy. Different VLPs formulations have been engineered for delivery of either Cas9 mRNA or Cas9 protein-guide RNA complex (Cas9 RNP). For production of Cas9 mRNA VLPs, an aptamer motif is appended downstream of the Cas9 coding sequence, and the Gag protein is expressed as a tandem fusion with the cognate aptamer binding protein (ABP) [77–83]. Whereas for production of Cas9 RNP VLPs, either the Cas9 is expressed as a fusion protein with Gag, or the aptamer motif is included within the sgRNA scaffold. The proof-of-principle for in vivo delivery of CRISPR components have been obtained in several studies (Table 1; ref [84–104]).

**Table 1:** Examples of use of non-viral and viral systems for in vivo delivery of CRISPR based therapeutics

Non-viral vectors				
**Delivery Method**	**Form of Cas Effector/Guide RNA**	**Targeted Genetic Element-Type of Editing**	**Cancer Model**	**Reference**
**Polymeric nanoparticles**				
Polyethylene glycol (PEG)- poly(lactic-co-glycolic acid) (PLGA) based cationic lipid-assisted polymeric nanoparticles (CLANs)	All-in-one Cas9-guide RNA plasmid	BCR-ABL gene knockout	Chronic myeloid leukemia (CML) mouse model	[84]
*A*cid-*r*esponsive *p*olycation-*f*luorinated (ARP-F) nanoparticles	All-in-one Cas9-guide RNA plasmid	BIRC5 gene knockout	A549 (lung cancer) xenografts in nude mice	[85]
Core–shell nanoparticles; core – cationic polyplex of plasmid and phenylboronic acid (PBA)-modified low molecular weight (LMW) polyethylenimine (PEI), shell – 2,3-dimethylmaleic anhydride (DMMA)-modified PEG-b-polylysine	All-in-one plasmid expressing dCas9-VP64 and guide RNA	CRISPRa of *miR-524*	MDA-MB-231 (breast cancer) xenografts	[86]
Hyaluronic acid (HA) decorated CP/Ad-SS-GD [Disulfide-bridged biguanidyl adamantine (Ad-SS-GD) complexed with β-cyclodextrin-conjugated low-molecular-weight polyethyleneimime (CP)]	Cas9 protein, guide RNA (Cas9 RNP)	KRAS (mutant) gene knockout	SW480 (colorectal adenocarcinoma) xenografts in nude mice	[87]
**Liposomes and lipid nanoparticles**				
Exosomes	Separate Cas9 and guide RNA plasmids	PARP1 gene knockout	SKOV3 (ovarian cancer) xenografts in nude mice	[88]
Internalizing RGD and cell penetration peptide (mHph3) coated PEI hydrogel-core 1,2-dioleoyl-3-trimethylammonium propane (DOTAP) liposomes	Cas9 protein and sgPLK1 plasmid	PLK1 gene knockout	U87 (glioma) xenografts	[89]
Folate receptor-targeting DOTAP liposomes	All-in-one Cas9-guide RNA plasmid	DNMT1 gene knockout	SKOV-3 (ovarian cancer) xenografts in nude mice	[90]
Micelles of quaternary ammonium-terminated poly(propylene oxide) (PPO-NMe3) and amphiphilic Pluronic F127	All-in-one Cas9-guide RNA plasmid	E7 gene knockout	HeLa xenografts in nude mice	[91]
R8-dGR (dual targeting cell penetrating peptide) modified liposomes	Separate Cas9 and guide RNA plasmids	HIF1A gene knockout	BxPC3 (pancreatic cancer) xenografts in nude mice	[92]
PEGylated liposomes	All-in-one Cas9-guide RNA plasmid	HPV16-E7/HPV18-E7 gene knockout	CasKi/HeLa (human papilloma virus 16- and 18-positive respectively) cervical cancer xenografts in Rag1 mice	[93]
Near-infrared (NIR) irradiation sensitive micelles	Cas9 protein, guide RNA (Cas9 RNP)	NRF2 gene knockout	CNE-2 (nasopharyngeal carcinoma) xenografts in nude mice	[94]
Microvesicles	Cas9 protein, guide RNA (Cas9 RNP)	IQGAP1 gene knockout	HepG2 (liver cancer) xenografts in nude mice	[95]
Extracellular vesicles	Cas9 protein, guide RNA (Cas9 RNP)	WNT10B gene knockout	HepG2 (liver cancer) xenografts in nude mice	[96]
Cyclic lipid nanoparticles (different formulations)	5-methoxyuridine modified Cas9 mRNA, modified sgRNA	PLK1 gene knockout	OV8 (ovarian cancer) xenografts in nude mice	[97]
**Gold nanoparticles**				
Core–shell nanoparticles; core – cationic TAT peptide modified gold nanoparticle with Cas9 protein and guide RNA plasmid, shell – cationic liposomes composed of DOTAP, 1,2-dioleoyl-sn-glycero-3-phosphoethanolamine (DOPE) and cholesterol, modified with 1,2-distearoyl-sn-glycero-3-phosphoethanolamine (DSPE)-PEG	Cas9 protein and sgPLK1 plasmid	PLK1 gene knockout	A375 (melanoma) xenografts in nude mice	[98]
**Porous nanoparticles**				
Single stranded DNA complementary to guide RNA, coated with PEI	Cas9 protein, guide RNA	Reporter gene knockout	U2OS-EGFP xenografts	[99]
Hollow mesoporous silica nanoparticles	All-in-one Cas9-guide RNA plasmid	EGFR gene knockout	H22 isografts in Kunming mice	[100]
**Viral vectors**				
**Adenovirus**		EGFR gene knockout	H1975/A549 (non-small cell lung cancer) xenografts in nude mice	[101]
		CRISPRa of DKK3 gene	PC3 (prostate cancer) xenografts in nude mice	[102]
**Adeno-associated virus**		HPV16 E6 and E7 gene knockout	HPV16 + anal cancer PDX in NOD SCID mice	[103]
**Lentivirus**		HIF1A gene knockout	SMMC-7721 (liver cancer) xenografts in nude mice	[104]

Different delivery systems (both non-viral and viral) have been used to ferry CRISPR components in vivo. These systems have been successfully used to affect gene knockout, transcriptional activation in robust fashion

### Optimization of CRISPR-Cas systems for In Vivo delivery

Several concerns pertaining to the gene therapy applications of CRISPR Cas systems have been addressed in the recent years. There have been developments towards rational miniaturization of the existing first-generation systems as well as characterization of new systems with more compact effector protein. A miniature CRISPR-Cas system, called as CasMINI has been engineered from the naturally occurring class 2 type V-F system, Cas12f (Cas14) [105]. Interestingly, the Cas effector protein of this system is much smaller than either Cas9 or Cas12a. Although natural system is not capable of performing gene editing in the mammalian cells, the CasMINI system, which evolved by means of both guide RNA and iterative protein engineering, has activity equivalent to Cas12a system. Different versions, which are products at different stages of iterative protein engineering, of the nuclease active CasMINI system exist, and they exhibit differential gene editing capabilities. Nuclease dead CasMINI (dCasMINI) system has also been created. The Cas effector of the CasMINI system has been fused with transcription activation or base editing module. The dCas-MINI-VPR and dCas9MINI-deoxyadenosine deaminase TadA systems have been shown to affect robust transcription activation and base editing at both reporter and endogenous gene loci respectively. The CasMINI-based systems become obvious choice for gene therapy applications as either the nuclease active effector or the dead CasMINI fusions that can effortlessly be packaged in adenoviral delivery systems. The coding sequences of dead CasMINI fusions (transcriptional activators, repressors, and base editors) are smaller than the AAV packaging limit. These systems promise to do away with the constraint imposed by the AAV payload limit for in vivo delivery. A Cas9 ortholog from *Campylobacter jejuni* (CjCas9) has been characterized [106]. It is one of the smallest Cas9 orthologs characterized till date with a coding sequence of only 2.95 kb. Using a nuclease mutant of this protein, a multi-component and single component transcription activation have been developed. In the multicomponent system, the dCjCas9-SunTag or SunTag-dCjCas9 and SunTag binding antibody fused to VPR module are expressed separately. Whereas in the single component system, the dCjCas9 has been fused to a truncated VPR module. The latter is also known as MiniCAFE system. Both these systems have been shown to activate gene expression at endogenous loci in both in vitro and AAV mediated delivery based in vivo models. Many CRISPR effectors, which are smaller as compared to the Cas9 and Cas12a enzymes, have been discovered recently. Two such effectors CasX (Cas12e, 986 amino acids) [107] and Casϕ (Cas12j, 700–800 amino acids) [108] were characterized by Doudna and her group. CasX belongs to the type V subtype E. Casϕ was characterized from the huge phages and belongs to type V subtype J. Whereas several other class 2 type V-F Cas12f1 orthologs have been characterized from *Acidibacillus sulfuroxidans* (AsCas12f1, 422 amino acids) [109], *Syntrophomonas palmitatica* (SpCas12f1, 497 amino acids) [110], and uncultured archaeon (Un1Cas12f1, 529 amino acids) [111, 112]. These Cas12f1 orthologs are attractive scaffolds for design of CRISPRa/i effectors and epigenetic/base editors to be delivered by vectors with payload restriction like AAVs.

Several Cas9 and Cas12a enzymes have been engineered to address off-target activity, PAM requirement and DNA cleavage activity. In vivo applications of Cas enzymes ideally demand no off-target activity and broader target range. SpCas9-high fidelity 1 (HF1) and “enhanced specificity” SpCas9 are two variants with lesser off-target activity [113, 114]. “Enhanced activity” FnCas12a (eaFnCas12a) is a variant that recognizes more flexible PAMs and higher cleavage activity [115]. Enhanced AsCas12a (enCas12a) has less stringent PAM requirement [116]. Whereas enAsCas12a-high fidelity 1 (HF1) is a variant that has qualities of enAsCas12a along with reduced off-target effects [116].

## Conclusions

The translation of CRISPR-based tools into standard-of-care therapeutic regimen can be achieved by addressing the concerns associated with the biology of CRISPR like, on-target cleavage efficiency, off-target effects, and delivery modalities for day-to-day clinical management of cancer.

For reliable use of CRISPR Cas genome-wide or subpool screens for identification of therapeutic targets and synthetic lethal gene pairs, optimized Cas proteins and guide RNA libraries are required. Recent studies have resulted in evolution of a Cas12a based combinatorial genetic screening toolkit comprised of two separate systems [64, 65]. These systems have been developed by harnessing the Cas12a’s intrinsic RNase activity that allows it to process crRNA array. CRISPR RNA expression cassette has also been modified by introduction of variant direct repeat (DR) sequence. This minimizes the risk of recombination-mediated collapse of the backbone without compromising Cas12a’s ability to produce multiple guide RNAs from the crRNA array. These libraries perform as good as the Cas9 based libraries at a much-decreased size. The latter’s application in combinatorial screening suffers from the fact the guide RNAs are expressed from separate promoters which compromises the stability of the guide RNA backbone. Less stringent PAM requirement and better on-target efficiency of second generation Cas12a nucleases also advocate their use in dual/multiple gene perturbation screens. Cas12a based libraries thus prove to be great tools for cases where starting biological material is limiting. Future studies would address better optimization of these libraries.

**Fig. 4:**
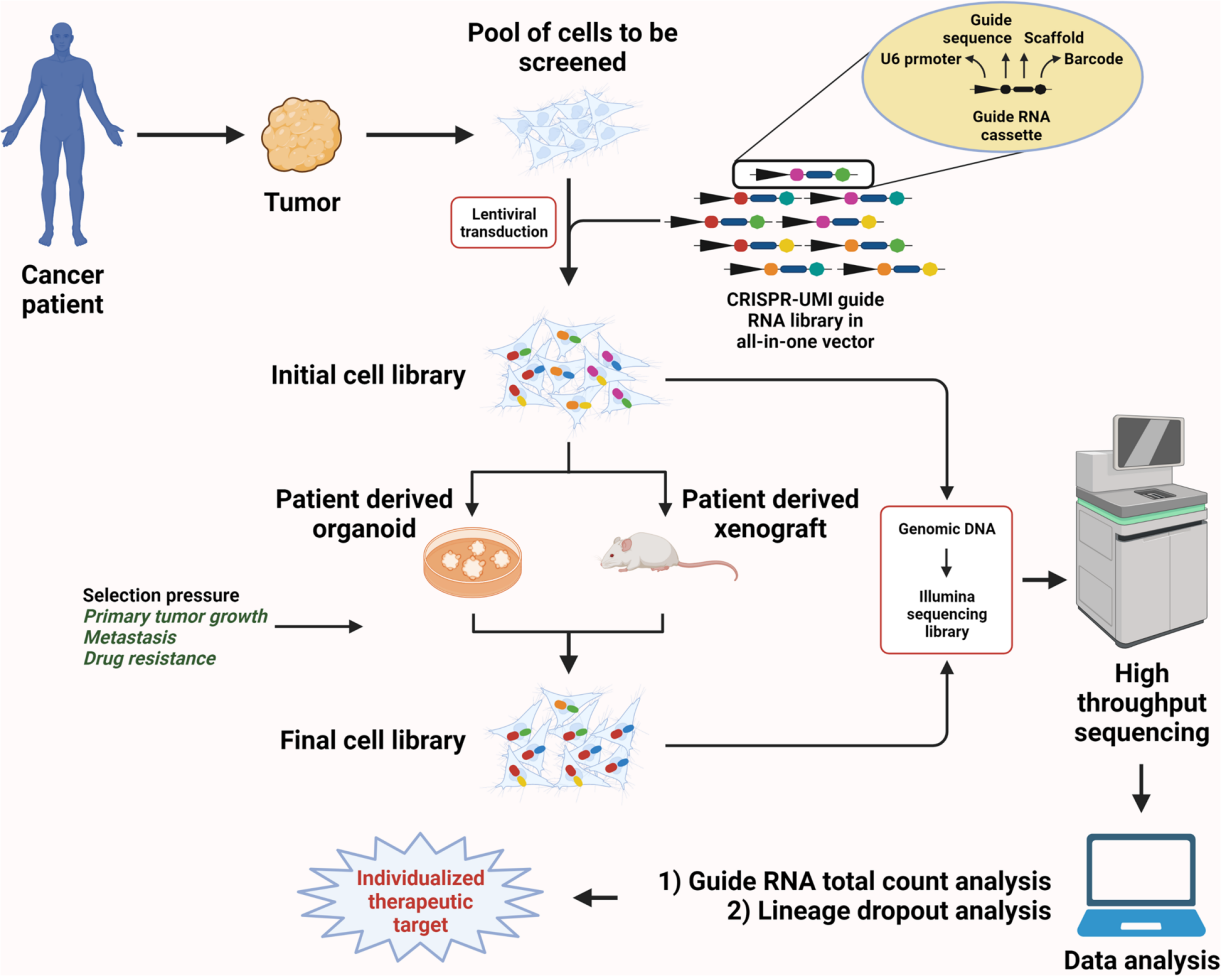
Workflow of optimized CRISPR-unique molecular identifier (UMI) pooled screen for identification of cancer patient-specific therapeutic targets. Ex vivo (patient derived organoid-based) or in vivo (patient derived xenograft-based) CRISPR screens are performed with barcoded guide RNA libraries for identification of individualized therapeutic targets with greater statistical power as these screens address the aspect of random clonal drift in the cell population that is being assayed. Created with BioRender.com with granted permission and license

Although loss-of-function (CRISPRko & CRISPRi) and gain-of-function (CRISPRa) screens have been used to identify causal genetic elements of different cancer phenotypes, not many have strived to understand the impact of the initial heterogeneity of cell clones on the outcome of the screen. Many genome-wide CRISPR screens have been performed with incorporation of barcodes in the guide RNA library to trace behavior of cell clones. Few recent studies attempted CRISPR knockout screen with concomitant clone tracing [117–120]. Guide RNA-barcode combinations are generated by inserting the latter either outside or within the guide RNA scaffold and acts as unique molecular identifiers (UMIs). As these CRISPR-UMI screens can statistically normalize the contribution of clone dynamics on relative abundance of guide RNAs, the approach is more suitable to address the underlying heterogeneity of cancer phenotypes. Clinically relevant CRISPR screens with PDOs and PDXs should exploit these gRNA-UMI libraries to ascertain genetic elements for therapeutic intervention (Fig. 4).

Several delivery options exist for transport of Cas9 plasmid or RNPs, their limitations or disadvantages are also manifold. These constraints should be addressed for inclusion in CRISPR based gene therapy clinical trials. Although the IDLVs offer greater safety than ICLVs in terms of transgene delivery, these exhibit less efficacy. Efforts would have to made for increasing efficacy of IDLVs for gene delivery.

Although AAVs hold great promise as gene therapy vectors, but the clinical translation is impeded by the neutralizing antibody generated against them by the human immune system. There have been recent efforts to map the epitopes in AAV serotypes which elicit neutralizing antibody response [121, 122]. More of these studies are required to create a global catalogue of neutralizing epitopes across AAV serotypes. This would help in evolution of next generation of immunologically inert, stealth rAAV vectors.

Application of VLPs for *in vivo* delivery is potentially restricted by the fact that these particles are prone to destruction by complement system or innate immune cell-mediated phagocytosis. VLPs can be stabilized in the circulation by enforcing high surface content of complement regulator CD55 or phagocytosis inhibitor CD47 [123, 124]. Although currently the field of CRISPR based precision medicine is in its infancy, the coming years would see greater translation of these tools to clinics.

## Data Availability

Not applicable.

## Abbreviations

CRISPR: Clustered regularly interspaced short pallindromic repeat
Cas: CRISPR-associated protein
DSB: Double strand break
Indel: Insertion-deletion
NHEJ: Non-homologous end joining
HDR: Homology directed repair
crRNA: CRISPR RNA
tracrRNA: Trans-activating CRISPR RNA
PAM: Protospacer adjacent motif
sgRNA: Single guide RNA
dCas9: Dead Cas9
CRISPRa: CRISPR activation
CRISPRi: CRISPR interference
SAM: Synergistic activation mediator
VPR: VP64-p65-Rta
GCN4: General control nonderepressible 4
TSS: Transcription start site
ddCas12a: DNase dead Cas12a
ZFN: Zinc finger nuclease
TALEN: Transcription activator-like effector nuclease
LV: Lentivirus
NGS: Next generation sequencing
ATAC-seq: Assay for transposase accessible chromatin using sequencing
PDO: Patient derived organoid
PDX: Patient derived xenograft
RNP: Ribonucleoprotein
NP: Nanoparticle
AAV: Adeno-associated virus
IDLV: Integration deficient lentivirus
ICLV: Integration competent lentivirus
VLP: Virus like particle
CasMINI: Miniature Cas
UMI: Unique molecular identifier
